# Prediction and Analysis *in silico* of Genomic Islands in *Aeromonas hydrophila*

**DOI:** 10.3389/fmicb.2021.769380

**Published:** 2021-11-29

**Authors:** Antonio Camilo da Silva Filho, Jeroniza Nunes Marchaukoski, Roberto Tadeu Raittz, Camilla Reginatto De Pierri, Diogo de Jesus Soares Machado, Cyntia Maria Telles Fadel-Picheth, Geraldo Picheth

**Affiliations:** ^1^Department of Clinical Analysis, Federal University of Parana, Curitiba, Brazil; ^2^Department of Bioinformatics, Professional and Technical Education Sector, Federal University of Parana, Curitiba, Brazil; ^3^Department of Biochemistry and Molecular Biology, Federal University of Parana, Curitiba, Brazil

**Keywords:** *Aeromonas hydrophila*, genomic island, virulence, metabolism, antibiotic resistance

## Abstract

Aeromonas are Gram-negative rods widely distributed in the environment. They can cause severe infections in fish related to financial losses in the fish industry, and are considered opportunistic pathogens of humans causing infections ranging from diarrhea to septicemia. The objective of this study was to determine *in silico* the contribution of genomic islands to *A. hydrophila*. The complete genomes of 17 *A. hydrophila* isolates, which were separated into two phylogenetic groups, were analyzed using a genomic island (GI) predictor. The number of predicted GIs and their characteristics varied among strains. Strains from group 1, which contains mainly fish pathogens, generally have a higher number of predicted GIs, and with larger size, than strains from group 2 constituted by strains recovered from distinct sources. Only a few predicted GIs were shared among them and contained mostly genes from the core genome. Features related to virulence, metabolism, and resistance were found in the predicted GIs, but strains varied in relation to their gene content. In strains from group 1, O Ag biosynthesis clusters OX1 and OX6 were identified, while strains from group 2 each had unique clusters. Metabolic pathways for myo-inositol, L-fucose, sialic acid, and a cluster encoding QueDEC, tgtA5, and proteins related to DNA metabolism were identified in strains of group 1, which share a high number of predicted GIs. No distinctive features of group 2 strains were identified in their predicted GIs, which are more diverse and possibly better represent GIs in this species. However, some strains have several resistance attributes encoded by their predicted GIs. Several predicted GIs encode hypothetical proteins and phage proteins whose functions have not been identified but may contribute to *Aeromonas* fitness. In summary, features with functions identified on predicted GIs may confer advantages to host colonization and competitiveness in the environment.

## Introduction

The bacterial genome is composed of a core genome containing the genetic information required for essential functions, and a flexible gene pool, which encodes additional traits that can be beneficial under certain circumstances. The flexible gene pool represents variable chromosomal regions and includes mobile and accessory genetic elements, such as bacteriophages, plasmids, insertion sequences, and genomic islands (GIs) (Dobrindt et al., 2004).

Genomic islands are syntenic blocks of accessory genes acquired by horizontal gene transfer (HGT) which contribute to the diversification and adaptation of microorganisms and offer a selective advantage for host bacteria. According to their gene content, GIs are described as pathogenicity, resistance, symbiosis, metabolic or fitness islands (Weinstock et al., 2000; Juhas et al., 2009; Rao et al., 2020). Most GIs are relatively large segments of DNA, usually between 10 and 200 kb, detected by comparisons among closely related strains; however, regions with sizes < 10 kb have also been identified and named genomic islets (Hacker et al., 1990; Juhas et al., 2009; Li and Wang, 2021). GIs usually differ from chromosomes in terms of GC content, tetranucleotide frequency, and codon usage. They are often flanked by small directly repeated (DR) sequences and are often inserted into tRNA genes. They can carry functional or cryptic genes encoding integrases, factors related to plasmid conjugation, phages involved in GI transfer, and transposons or insertion sequence (IS) elements, which may be implicated in mobilizing genetic material onto or deleting DNA from GI (Buchrieser et al., 1998; Juhas et al., 2009; Partridge et al., 2018). Currently, bioinformatics tools developed for GI prediction (Vernikos and Parkhill, 2006; Pundhir et al., 2008; Che et al., 2014; Soares et al., 2016; Wei et al., 2016; Bertelli et al., 2017) are available for the study of these mobile elements.

*Aeromonas* are Gram-negative bacteria ubiquitous in aquatic environments and can be isolated from virtually every environmental niche where bacterial ecosystems exist (Janda and Abbott, 2010). There are 36 species recognized of *Aeromonas* (Fernández-Bravo and Figueras, 2020), of which *A. hydrophila* is the most studied and the first species of the genus to have a sequenced genome (Seshadri et al., 2006; Grim et al., 2014; Rasmussen-Ivey et al., 2016b). *A. hydrophila* has been isolated from fresh and marine waters, diseased fish, poikilothermic aquatic animals, and warm-blooded animals (Martin-Carnahan and Joseph, 2005). In fish, *A. hydrophila* can cause a variety of diseases, including septicemia, red sore disease, and ulcerative infections, and has been linked to fish death worldwide, resulting in great economic losses (Janda and Abbott, 2010; Hossain et al., 2013, 2014; Rasmussen-Ivey et al., 2016a). In humans, *A. hydrophila* is associated with both intestinal and extraintestinal diseases (Martin-Carnahan and Joseph, 2005), such as gastroenteritis, wound infection, septicemia, pneumonia, soft tissue infections, peritonitis, hepatobiliary tract infections, and necrotizing fasciitis (Janda and Abbott, 1998, 2010; Grim et al., 2014).

Genome sequencing and comparative genomic analysis have allowed major advances in the study of *Aeromonas* virulence. Highly virulent strains recovered from humans or fish have been identified, several genes have been associated with pathogenicity and some are related to GIs in some strains (Seshadri et al., 2006; Hossain et al., 2013; Grim et al., 2014; Pang et al., 2015; Rasmussen-Ivey et al., 2016a). The objective of this study was to analyze *A. hydrophila* genomes using a GI predictor, determine the distribution of predicted GIs, their characteristics, functions encoded, core genome content, and sharing among strains.

## Materials and Methods

### *Aeromonas hydrophila* Genomes

The complete genomes of *A. hydrophila* available at the National Center for Biotechnology Information database (Geer et al., 2010) as at 12/01/2019 were used in this study. The source and genome characteristics of the *A. hydrophila* strains are shown in Table 1.

**TABLE 1:** General characteristics of *Aeromonas hydrophila.*

Strain	RefSeq ID	Genome Size (Mb)	Genome GC%	Gene number	Source	Collection Year	Country
*A*. *hydrophila* ATCC 7966	NC_008570.1	4.74	61.50	4,283	Canned milk	No info	United States
*A*. *hydrophila* AL06-06	NZ_CP010947.1	4.88	61.40	4,572	Goldfish	2006	United States
*A*. *hydrophila* AHNIH1	NZ_CP016380.1	4.91	61.20	4,551	Perirectal swab	2013	United States
*A*. *hydrophila* ML09-119	NC_021290.1	5.02	60.80	4,646	Catfish	2009	United States
*A*. *hydrophila* AL09-71	NZ_CP007566.1	5.02	60.80	4,649	Catfish	2009	United States
*A*. *hydrophila* pc104A	NZ_CP007576.1	5.02	60.80	4,648	Pond soil	2010	United States
*A*. *hydrophila* NJ-35	NZ_CP006870.1	5.28	60.50	4,902	Diseased carp	2010	China
*A*. *hydrophila* JBN2301	NZ_CP013178.1	5.13	60.80	4,767	Crucian carp	2009	China
*A*. *hydrophila* J-1	NZ_CP006883.1	5.00	60.90	4,616	Diseased carp	1989	China
*A*. *hydrophila* GYK1	NZ_CP016392.1	4.95	60.80	4,534	Siniperca chuatsi	2001	China
*A*. *hydrophila* D4	NZ_CP013965.1	5.10	60.80	4,729	Megalobrama amblycephala	2012	China
*A*. *hydrophila* strain ZYAH75	NZ_CP016990.1	4.96	61.30	4,598	Wound secretion	2015	China
*A*. *hydrophila* strain ZYAH72	NZ_CP016989.1	5.16	60.70	4,797	Crucian carp	2015	China
*A*. *hydrophila* AH10	NZ_CP011100.1	4.91	61.10	4,561	Grass carp	2011	China
*A*. *hydrophila* WCHAH045096	NZ_CP028568.1	5.02	61.10	4,691	Sewage	2015	China
*A*. *hydrophila* MX16A	NZ_CP018201.1	4.78	61.60	4,445	Water	2012	China
*A*. *hydrophila* 4AK4	NZ_CP006579.1	4.52	62.00	4,164	Raw Sewage	1999	China
*A*. *hydrophila* YL17	NZ_CP007518.2	4.80	61.60	4,392	Compost	No info	Malaysia
*A*. *hydrophila* KN-Mc-1R2	NZ_CP027804.1	4.91	61.00	4,629	*Myocastor coypus*	2016	South Korea

### Phylogenetic Analysis

A phylogenetic tree was generated using the software Spaced Words Projection – SweeP (De Pierri et al., 2020) with the complete genomes of all *Aeromonas* species available from the NCBI database. Default SweeP criteria were used in the analysis. Data regarding the organisms used are shown in Supplementary Table 1.

### Genomic Islands Prediction and Analysis

IslandViewer 4 (Bertelli et al., 2017) was used to predict GIs in *A. hydrophila* strains. GIs < 10 kb were considered genomic islands unless otherwise stated. Some GIs predicted by SIGI-HMM (Waack et al., 2006) and IslandPath-DIMOB (Hsiao et al., 2003) two components of IslandViewer 4, were partially superimposed, increasing the number of predictions. Then, a verification of these regions was conducted to determine the start and end of the GIs manually, avoiding misidentification in the number of predicted GIs. After the identification of each predicted GI by IslandViewer 4, the regions were selected and visualized using Artemis software (Rutherford et al., 2000) to generate .GBK and .FASTA files for each GI. These files contained the amino acid sequence, the name, and the protein ID of each product of each GI, and were used to create multi-fasta files for each microorganism. These files were submitted to RAST (Brettin et al., 2015) for standardization of gene nomenclature. The IslandViewer version 4.0 (Web Server) was used.

### Tools and Databases for Traits Prediction

To determine the traits encoded by the predicted GIs, datasets containing the amino acid sequences of the GIs of each *A. hydrophila* strain were compared with the amino acid sequences of the databases indicated below using the online web server CD-HIT Suite: Biological Sequence Clustering and Comparison (CD-HIT-2D; version v4.8.1-2019-0228) (Huang et al., 2010). The minimum threshold for sequence identity cut-off was 70%. All other parameters of CD-HIT-2D are indicated in Supplementary Table 2.

The following databases were used to identify traits related to virulence, antibiotic resistance, or drug targets encoded on the predicted GIs:

Pathosystems Resource Integration Center – PATRIC (Wattam et al., 2017). The virulence factor database – VFDB (Liu et al., 2019). PHIDIAS: Pathogen-host interaction data integration and analysis system VICTORS (Sayers et al., 2019). The Comprehensive Antibiotic Resistance Database CARD (Jia et al., 2017). National Database of Antibiotic Resistant Organisms^1^. DrugBank (Wishart et al., 2018). Therapeutic Target Database (TTD) (Li et al., 2018). For the identification of metabolic traits, KEGG Mapper (Kanehisa and Sato, 2020) was used with family/genus 642 ID. The data obtained from the tests were manually curated.

### Phage Prediction

Phage regions were analyzed using PHASTER Phage Search Tool Enhanced Release (Arndt et al., 2016). The PHASTER search phages regions using BLAST through file analysis in .GBK or .FASTA format. The sequences are compared with the NCBI Phage database and a phage database developed by Srividhya et al. (2006). Phage-like genes are then grouped into phage regions using DBSCAN (Ester et al., 1996). Regions corresponding to phages are assigned scores and colors depending on the level of identity and coverage of the region. Intact phage (> 90 score) are colored green; questionable phage (70-90 score) labeled blue; incomplete phage (< 70 score) are colored red. For this analysis, complete genomes of *A. hydrophila* were submitted to PHASTER to search for phage regions, and the results were compared with predicted GIs for identification of phage-containing GIs.

### Shared Genomic Islands

To compare the predict GIs, genes of each GI were concatenated and used to create individual multi-fast files for each strain. After this step, BLASTp was used to infer homology, considering a minimum of 75% of identity and sequence coverage for comparisons between single GIs against all other predicted GIs in all strains. Power Bi, a software for generating interactive reports with data modeling tools, and chord diagrams were used to represent the proportion of GIs shared among the strains. An image was generated using the data obtained from the total number of shared GIs. The nodes were organized around a circle, with the proportion between points connected to each other, represented proportionally by the size of each arc^2^. Power Bi version 2.96.1061.0 (Windows 64-bit) was used in this study.

### Core Genome and Related Genomic Islands

The core genome of *A. hydrophila* strains was determined using the web server EDGAR version 3.0 (Web Server) https://edgar3.computational.bio.uni-giessen.de/ (Dieckmann et al., 2021). This bioinformatics tool is based on ‘score ratio values’ (SRVs) methodology for orthology estimation. To calculate the SRVs, all BLAST scores are normalized against the maximum score, which is the self-hit of a query sequence. Then, the distribution of the SRVs is analyzed to derive a threshold that is tailored to the data, usually around an SRV of 0.3 (30%). Furthermore, an initial e-value cutoff of 1e-05 is used. This orthologous approach is such that only one-to-one pairs are found. For duplicated genes and paralogs, a single hit is found, and additional copies are missed. Pseudogenes are excluded from these analyses. Core genome calculation requires one reference genome, for which *A. hydrophila* ATCC 7966 was chosen. Results from the core genome (EDGAR) are presented with a locus tag for each gene and strain with the amino acid sequence. After identifying the genes from the core genome, they were compared with predicted GIs using CD-HIT, a tool developed for clustering very long microbial genomic sequences. In this analysis the comparison was performed gene-by-gene for all GIs of each strain using amino acid sequences. The cut-off rate used was 90% identity.

### Cluster Alignments

Mauve, a tool for multiple genome alignments that can be used to view genomic rearrangements, inversions, and large insertions in related genomes, was used to analyze O antigen clusters. The alignment is shown as an image formed by colored blocks, each one of them representing genome region aligned and internally free of genomic rearrangements (Darling et al., 2004). Mauve version 2.4.0 (Windows 64-bit) was used in this study.

### Statistical Methods

Variables with normal distributions (Kolmogorov-Smirnov) were reported as mean ± 1-standard deviation. Comparison among groups were tested with Student *t*-test (2-tailed) or by one-way ANOVA for multiple groups comparisons. A *P*-value < 0.05 was considered significant.

All calculations were performed using MedCalc version 17.6 (MedCalc Statistical Software bvba, Ostend, Belgium).

## Results

### Phylogenetic Analysis and General Characteristics of *Aeromonas hydrophila* Strains

**FIGURE 1:**
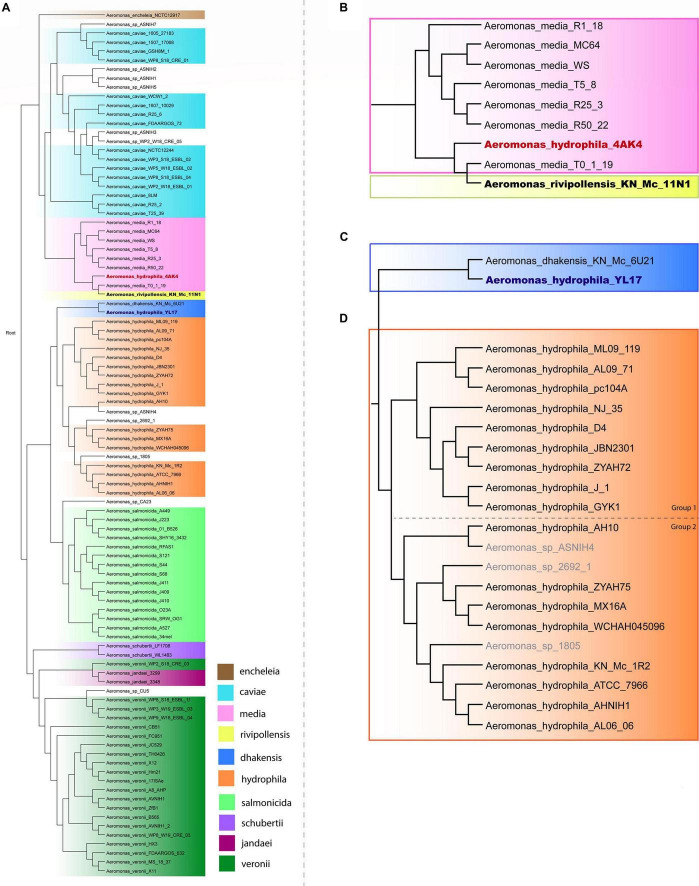
Phylogenetic tree of the *Aeromonas* species. **(A)** Phylogenetic tree generated from the whole genome of 96 *Aeromonas* strains. **(B)** Highlighted branch of “*A. hydrophila* 4AK4.” **(C)** Highlighted branch of “*A. hydrophila* YL17.” **(D)** Highlighted branch of *A. hydrophila*; *s*trains analyzed are subgrouped into two main subclades, separated by dotted lines: ML09-119, AL09-71, pc104A, NJ-35, D4, JBN2301, ZYAH72, J-1, and GYK1 (group 1); AH10, ZYAH75, MX16A, WCHAH045096, KN-Mc-1R2, ATCC 7966, AHNIH1, and AL06-06 (group 2).

A phylogenetic tree based on the complete genome of *Aeromonas* species was generated to verify the relationships among *A. hydrophila* isolates. A total of 96 genomes from 10 *Aeromonas* species and some strains identified only to the genus level were included in this analysis, the results of which are shown in Figure 1. Strains of *A. schubertii*, *A. veronii*, *A. salmonicida*, *A. dhakensis*, *A. hydrophila*, *A. encheleia*, *A. media*, *A. rivipollensis*, *A. jandaei*, and *A. caviae* were separated into distinct branches of the phylogenetic tree according to their species. However, there were some exceptions, with *A. veronii* WP2_S18_CRE03 grouped with *A. jandaei* strains 3299 and 3348; *A. hydrophila* YL17 grouped together *A. dhakensis* KN_Mc_6U21, and *A. hydrophila* 4AK4 with *A. media* T0-1-19 and *A. rivipollensis* KN_Mc_11N1, suggesting that these strains were misidentified at the species level. Thus, the genomes of strains YL17 and 4AK4 were removed from this study. Additionally, a small number of strains previously identified only at the genus level clustered together with certain species (Figure 1).

The 17 complete *A. hydrophila* genomes that were analyzed for predicted GIs differed in genome size, which varies by up to 0.54 Mb, as observed between strains ATCC 7966, which has the smallest genome (4.74 Mb) and NJ-35 (5.28 Mb) the largest; the GC content varied from 60.50% in strain NJ-35 to 61.60% in strain MX16A (Table 1).

Phylogenetic analysis classified the 17 *A. hydrophila* strains into two main groups (Figure 1). Group 1 contained nine strains, isolated in China or the United States, recovered from diseased fish, except for pc104A, which was recovered from the soil of a catfish pond (Table 1). The Chinese and American *A. hydrophila* strains were separated into different subclades of group 1. Group 2 contained strains recovered from different sources, including the environment (ATCC 7966, MX16A, and WCHAH045096), human clinical samples (ZYAH75, AHNIH1), *Myocastor* (KN-Mc-1R2), and diseased fish (AH10 and AL06-06). They were also separated into subclades according to their geographical origins.

Strains from groups 1 and 2 differ in relation to the mean genome size; 5,08 and 4,89 Mb, respectively. Interestingly, all strains of group 1 presented genomic GC contents below 61% (60.50% to 60.90%), while those of group 2 were 61% or higher (61.00 to 61.60%; Table 1), a significative difference (*P* < 0.001; *t*-test), however, stats were underpowered.

### Characteristics of Predicted Genomic Islands of *Aeromonas hydrophila* Strains

The number of predicted GIs varied widely among the strains, from 13 in *A. hydrophila* ATCC 7966 and MX16A to 33 in strain NJ-35, and the average size ranged from 12402 bp in AL06-06 to 29317 in JBN2301. A significant difference was observed in terms of the ratio between the total GI sequence size and host genome size, which varied from 4% in strain ATCC 7966 to 12% in NJ-35. The variation in GC content within GIs ranged from 34% to 66%, both in *A. hydrophila* isolate AH10 (Table 2). In general, the genomes of group 1 strains presented a higher average number of predicted GIs, genes in GIs, larger mean GI size, and ratio of GIs relative to genome size than those of group 2 (Table 2).

**TABLE 2:** General data of predicted genomic islands (GIs).

Strain	GIs^1^	> 10 kb^2^	< 10 kb^3^	A.SGIs^4^	Genes^5^	> GC%^6^	< GC%^7^	Av.GC%^8^	Gen.Siz^9^	GI Total Size^10^	Ratio%^11^
ATCC 7966	13	6	7	16279	204	63%	43%	51%	4744448	211630	4%
MX16A	13	7	6	17185	246	63%	39%	51%	4783504	223399	5%
ZYAH75	15	10	5	19549	401	59%	37%	51%	4955171	293237	6%
AH10	15	11	4	19391	314	66%	34%	51%	4908265	290871	6%
KN-Mc-1R2	16	7	9	20054	379	60%	39%	50%	4911246	320867	7%
AL06-06	19	9	10	12402	252	63%	38%	51%	4884823	235642	5%
AHNIH1	20	17	3	20042	404	65%	38%	51%	4906118	400837	8%
WCHAH045096	27	18	9	19387	556	63%	37%	52%	5022867	523460	10%
J-1 *	17	15	2	28323	504	58%	35%	48%	5000814	481498	10%
GYK1	19	16	3	25238	479	58%	35%	47%	4951765	479521	10%
JBN2301 *	19	17	2	29317	597	58%	35%	49%	5127362	557016	11%
ML09-119 *	21	18	3	24627	531	58%	35%	49%	5024500	517159	10%
AL09-71 *	22	18	4	23889	541	58%	35%	49%	5023861	525556	10%
ZYAH72	22	19	3	26601	626	58%	35%	49%	5159182	585215	11%
pc104A *	22	19	3	24085	546	58%	35%	49%	5023829	529868	11%
D4	23	18	5	23873	579	62%	35%	50%	5100520	549072	11%
NJ-35 *	33	20	13	19477	661	65%	35%	52%	5279644	642740	12%

Strains from phylogenetic group 1 are underlined; *hypervirulent A. hydrophila (vAh).

*^1^Total number of predicted GIs.*

*^2^Total GIs > 10,000 base pairs.*

*^3^Total GIs < 10,000 base pairs.*

*^4^Average size of GIs.*

*^5^Total genes in predicted GIs.*

*^6^Higher percentage of guanine and cytosine in GIs.*

*^7^Lower percentage of guanine and cytosine in GIs.*

*^8^Average percentage of guanine and cytosine in GIs.*

*^9^Genome size.*

*^10^Total size of GIs.*

*^11^Percentage of GI size in relation to genome size.*

Altogether, the data indicated differences in relation to genome size and GC content, distribution, and characteristics of GIs of strains from groups 1 and 2. Genes identified on the predicted GIs are listed in Supplementary Table 3.

### Functions Encoded by the Predicted Genomic Islands

Determination of the functions of genes in the predicted GIs was based on databases related to virulence, resistance, metabolism, drug targets, and RAST annotation. A complete list of genes with identified functions and the corresponding databases is shown in Supplementary Table 4. The main traits identified are described below.

#### Traits Related to Virulence

##### O antigen

**FIGURE 2:**
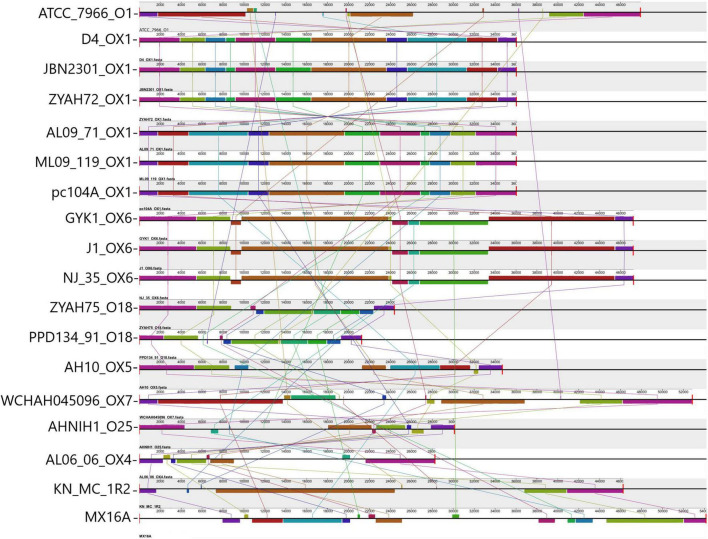
Comparison of O antigen biosynthesis clusters encoded in predicted genomic islands (GIs). Alignment of the O Ag cluster from 17 *A. hydrophila* strains analyzed and strain PPD134-91. Blocks with the same color indicate homologous regions. The scale indicates the size of the cluster. Strain designations and respective serogroups are indicated below the representation of the cluster. Serogroups identified OX1 (D4, JBN2301, ZYAH72, AL09-71, ML09-119, pc104A); OX6 (GYK1, J-1, NJ-35); O18 (comparison between PPD134/91 serogroup O18, and ZYAH75 cluster); OX5 (AH10); OX7 (WCHAH045096); O25 (AHNIN1); OX4 (AL06-06); O1 (ATCC 7966); not determined (KN-MC-1R2, MX16A). The O Ag cluster of strains pc104a, AL09-71, and ML09-119 are in the reverse orientation in the genome of the strains. The name of each strain and the respective serogroup are indicated in front of the blocks representing the O Ag cluster.

Gene clusters encoding proteins associated with the biosynthesis of O antigen (O Ag) were identified in the GIs of all *A. hydrophila* strains. Ten distinct O Ag clusters were found among the 17 strains analyzed in this study, including serogroups O1, O25, and putative serogroups OX1, OX4, OX5, OX6, and OX7 (Figure 2). The O Ag cluster of strain ZYAHA75 presented identity and query coverage of 89.69% and 97%, respectively, with serogroup O18 of *A. hydrophila* strain PPD134/91 compared in Figure 2. The O Ag cluster of KN-Mc-1R2 contains *cysN* and *cysD* genes encoding enzymes related to sulfur metabolism, and that of strain MX16A is interrupted by genes encoding transposases, suggesting that HGT played a role in the formation of this cluster. The comparison of the OAg cluster sequence of strain MX16A showed higher homology with that of *Aeromonas veroni* A8-AHP, with 77% of coverage and 93% of identity. When compared to the same species, better results were observed with *A. hydrophila* G5380, with 50% of coverage and 94% of identity. For KN-MC-1R2, better results were obtained with *Aeromonas hydrophila* 3924, with 44% of coverage and 98% of identity. When compared with another species, 41% of cover and 89% of identity was found with the cluster of *Aeromonas caviae* KAM345.

##### Flagella

Genes from polar flagella region 2 (*flaA flaBGHJ maf-1*) were identified in the predicted GIs of most strains, with additional genes found in several of them. Three types of predicted GI were observed according to the identity and number of genes encoded. One, approximately 7 kb encoding *flaA flaBGHJ maf-1* and a hypothetical protein was observed in strains ATCC 7966, AL06-06, and AHNIH1. Predicted GIs of approximately 19 kb encoding the polar flagella region 2 together with other genes, including a flagellin-like, transposase, *neuB*, and *flmD* (related to polar flagella glycosylation), were found in all strains from group 1 and in AH-10. Finally, GIs of approximately 15 kb containing the polar flagella region 2 genes, *neuB, flmD*, and other genes distinct from those of the GI described above, were identified in strains WCHAH045096 and MX16A. In strains KN-Mc-1R2 and ZYAH75, no GIs containing flagellar region 2 genes were predicted, although these regions are present in their genomes.

##### Components of Type VI Secretion System

Genes encoding VgrG and Hcp, components of the expelled puncturing structure of type VI secretion system (T6SS) were found in the predicted GIs of strains AH10, AL06-06, and KN-Mc-1R2. In the first two strains, VgrG-encoding genes are present in two predicted GIs.

Additionally, certain features that may potentially be associated with virulence are encoded in predicted GIs of some strains, including ankyrin proteins (AnkB), which are encoded together with catalase (KatE) and, in most of them, with super oxide dismutase; Fic (filamentation induced by cyclic AMP) proteins, of which two types PA0574 and KPN03553 were identified; pilin Flp; the zona occludens toxin and accessory cholera enterotoxin, were sequentially encoded (Table 3). Of the genes with identified functions, some were found in genomic islets, which contain low numbers of genes and are generally poorly explored or studied. Among the genes identified in genomic islets are those encoding Fic domain protein PA0574 (strain ZYAH72, GI-18); zone occludens toxin and accessory cholera enterotoxin (strains KN-Mc-1R2, GI-11, AHNIH1, GI-17), and VgrG (strains AH10, GI15; AL06-06, GI-16).

**TABLE 3:**
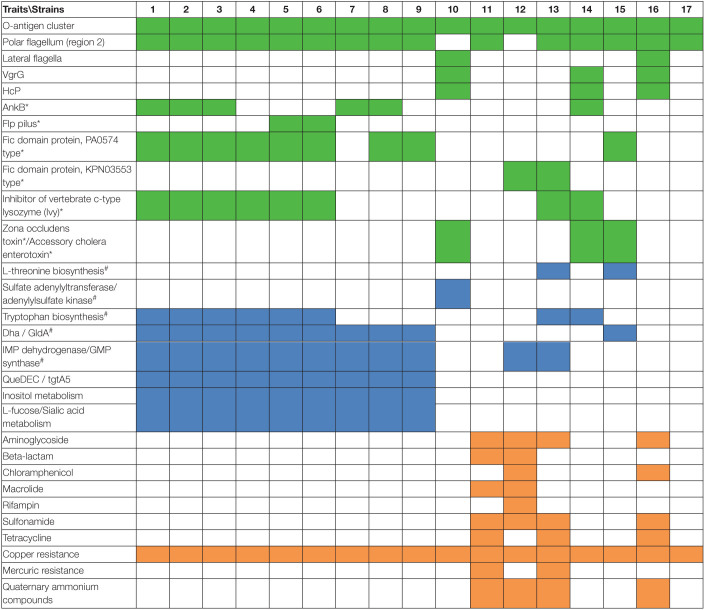
Main traits encoded in predicted genomic islands (GIs).

Colors indicate distinct classes of traits; green corresponds to virulence or *putative virulence genes; blue indicates metabolism; orange corresponds to resistance. ^#^Indicates core genome. Numbers in the header correspond to the strains (^1^ZYAH72, ^2^JBN2301, ^3^D4, ^4^ML09-119, ^5^AL09-71, ^6^pc104A, ^7^NJ-35, ^8^J-1, ^9^GYK1, ^10^KN-Mc-1R2, ^11^MX16A, ^12^ZYAH75, ^13^WCHAH045096, ^14^AH10, ^15^AHNIH1, ^16^AL06-06, ^17^ATCC 7966). AL06-06: Aminoglycoside 3″-nucleotidyltransferase - ANT(3″)-Ia; chloramphenicol O-acetyltransferase, CatB family; dihydropteroate synthase type-2 sulfonamide resistance protein; small multidrug resistance (SMR) efflux transporter - QacE delta 1, quaternary ammonium compounds; tetracycline resistance, MFS efflux pump – TetC. MX16A: Aminoglycoside 3″-nucleotidyltransferase - ANT(3″)-Ia; aminoglycoside N(3)-acetyltransferase - AAC(3)-II,III,IV,VI,VIII,IX,X; class A beta-lactamase - VEB family, extended-spectrum; dihydropteroate synthase type-2 sulfonamide resistance protein; macrolide 2′-phosphotransferase - Mph(A) family; SMR efflux transporter - QacE delta 1, quaternary ammonium compounds; tetracycline resistance, MFS efflux pump Tet(A); mercuric resistance operon (regulatory protein MerR; mercuric transport protein, MerT; periplasmic mercury(+ 2) binding protein, MerP; mercuric ion reductase; mercuric resistance transcriptional repressor, MerD; mercuric transport protein, MerE). WCHAH045096: Aminoglycoside 3″-nucleotidyltransferase - ANT(3″)-Ia; dihydropteroate synthase type-2 - sulfonamide resistance protein; small multidrug resistance (SMR) efflux transporter - QacE delta 1, quaternary ammonium compounds; tetracycline resistance, MFS efflux pump - Tet(A); mercuric resistance operon (regulatory protein MerR; mercuric transport protein, MerT; periplasmic mercury(+ 2) binding protein, MerP; mercuric ion reductase; mercuric resistance transcriptional repressor, MerD; mercuric transport protein, MerE). ZYAH75: Aminoglycoside 3″-nucleotidyltransferase ANT(3″)-Ia; aminoglycoside 3′-phosphotransferase APH(3′)-I; aminoglycoside 3″-phosphotransferase - APH(3″)-I; aminoglycoside N(6′)-acetyltransferase - AAC(6′)-Ib/AAC(6′)-II; aminoglycoside 6-phosphotransferase - APH(6)-Ic/APH(6)-Id; chloramphenicol resistance, MFS efflux pump - CmlA family; chloramphenicol/florfenicol resistance, MFS efflux pump - FloR family; class A beta-lactamase - CTX-M family, extended-spectrum; class A beta-lactamase - TEM family; class D beta-lactamase - OXA-10 family; dihydropteroate synthase type-2 - sulfonamide resistance protein; macrolide 2′-phosphotransferase - Mph(A) family; rifampin ADP-ribosyl transferase; SMR, efflux transporter - QacE, quaternary ammonium compounds.

#### Traits Related to Resistance

Genes associated with antibiotic resistance were restricted to the predicted GIs from strains AL06-06, ZYAH75, MX16A, and WCHAH045096. These genes were associated with resistance to sulfonamide, aminoglycoside, tetracycline, rifampin, chloramphenicol, and macrolides, in addition to TEM beta-lactamase, and distinct extended-spectrum beta-lactamases that are able to hydrolyze different beta-lactams.

Additionally, genes related to mercury, copper, and chromium compounds, and ammonium quaternary resistance were identified in the GIs of some strains (Table 3, Supplementary Table 4).

#### Traits Related to Metabolism

Genes or clusters of genes associated with metabolism were identified in the predicted GIs (Table 3).

Among them, the gene cluster *dhaKLM*, encoding subunits of dihydroxyacetone kinase (Dha), *gldA*, glycerol dehydrogenase (GldA), and *dhaR*, encoding the transcriptional regulator DhaR, are associated with glycerol metabolism.

Genes encoding the enzymes inosine-5′-monophosphate dehydrogenase (IMP dehydrogenase) and GMP synthase were also identified in the predicted GIs of several strains. They participate in purine metabolism in the synthesis of GMP from IMP.

Other predicted GIs contained genes related to amino acid biosynthesis. Enzymes that participate in the biosynthesis of tryptophan from chorismate are encoded by the predicted GIs from several strains. Additionally, genes encoding the bifunctional aspartate kinase/homoserine dehydrogenase I, homoserine kinase, and threonine synthase, enzymes that are part of the aspartate pathway of amino acid biosynthesis related to threonine biosynthesis, were identified in predicted GIs in a few strains.

The *cys*N and *cysD* genes encoding subunits of the enzyme sulfate adenylyltransferase, and *cysC* encoding adenylylsulfate kinase were found to be part of the predicted GIs of a certain strains. These enzymes participate in some steps in the process of assimilatory sulfate reduction, in which sulfate is reduced to hydrogen sulfide and then incorporated in cysteine and methionine biosynthesis.

Genes encoding 6-carboxytetrahydropterin synthase (QueD), 7-carboxy-7-deazaguanine synthase (QueE), and 7-cyano-7-deazaguanine synthase (QueC) were identified in several of the predicted GIs, and participate in the synthesis of queuosine.

Genes associated with carbohydrate metabolism, including sialic acid, fucose, and inositol metabolism, were also identified in the predicted GIs (Table 3).

#### Phages

PHASTER analysis indicated the presence of intact and questionable phages in strain genomes. For intact phages, the most common were *Aeromonas* phage phiO18P, *Salmonella* phage SEN8, *Escherichia* phage Lys12581Vzw, *Shigella* phage POCJ13, and for questionable phages *Escherichia* phage 520873 and *Shigella* phage POCJ13. Only strains ATCC 7966, GYK1, and MX16A did not have any phage-related regions with a score > 70. However, no intact phages were found on the predicted GIs, but several of them contained partial phage sequences (Supplementary Table 5).

#### Other Features Encoded in Predicted Genomic Islands

Several other features are encoded in predicted GIs, including proteins related to chemotaxis signal sensing/transduction, response regulators, and a two-component system; a type I restriction modification system, DNA repair proteins, transporters, toxin-antitoxin modules, cytochrome associated proteins, an inhibitor of vertebrate c-type lysozyme (Ivy), and flagella and pili biosynthesis-associated proteins. Finally, GIs encoding transposon-associated proteins, conjugation-related proteins, or almost exclusively hypothetical proteins, were also observed in *A. hydrophila.* The distribution of these features varied among strains (Supplementary Table 3).

## Predicted Genomic Islands Containing Genes From Core Genome

The presence of genes from the core genome was detected in several of the predicted GIs from all strains (Supplementary Table 3), which reached 13% (strain AH10) to 27% (strain AL06-06), with an average of 18% among the *A. hydrophila* isolates.

However, in a few islands, a high number of genes from the core genome were detected. This was observed mainly in the predicted GIs containing the gene cluster *dhaKLM gldA*, which were identified as part of the core genome. In addition, genes encoding ribosomal proteins and transcription-related factors, rRNAs, RNA polymerase subunit ββ′, and some tRNAs, among others, are present in the predicted GIs. The presence of repeat regions and transposases between these genes was also observed, which may have contributed to its identification as a GI. The predicted GI containing the cluster *scsABCD*, associated with copper resistance, also contains a high proportion of genes from the core genome.

Other genes from the core genome in predicted GIs encode the enzyme IMP dehydrogenase, enzymes associated with biosynthesis of tryptophan or threonine, and the enzyme adenylylsulfate kinase. These and other genes related to the core genome are highlighted in blue in Supplementary Table 3.

## Shared Genomic Islands

**FIGURE 3:**
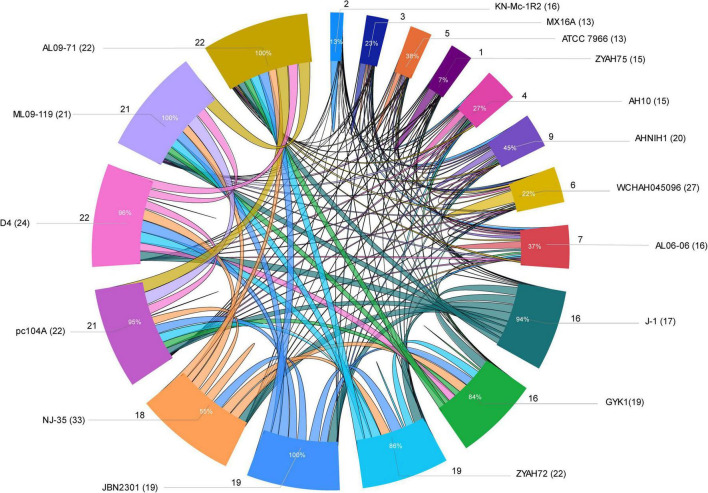
Sharing of predicted genomic islands (GIs) among *A. hydrophila* strains. Ratio of GIs shared among strains. Distribution of the data is presented as the total number of predicted GIs, the total number of shared GIs, and their related percentages. The circle indicates the predicted GIs shared proportionally by the size of each arc; the names of the strains are indicated outside the circle with the number of predicted GIs in parentheses; the number of GIs shared is indicated by the lines and the proportion inside the arc; lines connecting strains indicate sharing of predicted GIs.

Several strains share predicted GIs, as indicated by comparative analysis performed considering at least 75% amino acid sequence identity and coverage as criteria (Figure 3; Supplementary Table 6). Some of the predicted GIs were shared among most of the strains, while others were shared only among those of phylogenetic group 1 or group 2. Strains from group 1 shared all or most of their predicted GIs, while those from group 2 shared only a few (Figure 3; Supplementary Table 6).

The main predicted GIs with identified functions shared among two or more strains from both groups included those encoding ScsABCD, DhaKLM/GldA, and enzymes associated with tryptophan biosynthesis/Ivy. Of these, the first is the most widespread and is present in all strains except KN-Mc-1R2. This strain also contains the cluster ScsABCD; however, it is encoded in a larger GI that did not meet the criteria used in this study to define a shared island with other strains. The predicted GIs with characterized functions shared only among strains from group 1 included those encoding inositol and sialic acid/L-fucose metabolism; QueDEC, the O antigen cluster for putative OX1 or OX6 serogroups, and Fic PA0574 (Supplementary Tables 3,6). Additionally, predicted GIs encoding toxin/antitoxin systems, phages or hypothetical proteins are shared among them. In group 2, predicted GIs encoding the zona occludens toxin/accessory cholera enterotoxin and flagellar regions 1 and 2 were the most common shared GIs (Supplementary Tables 3,6).

## Discussion

In this study, complete genomes of *A. hydrophila* strains were analyzed using the Island Viewer 4 bioinformatics tool for GI prediction in order to determine the distribution of GIs and their contributions in such strains, however, while some of the features encoded have functions defined, others are putative and have to be experimentally demonstrated in *Aeromonas*. A phylogenetic analysis performed with SweeP software showed that two strains identified as *A. hydrophila* (4AK4 and YL17) were misidentified at species level. These findings are in agreement with the studies by Beaz-Hidalgo et al. (2015) and Moriel et al. (2021) which indicated several problems with the taxonomic affiliation of *Aeromonas* genomes deposited at the NCBI database. These findings show the relevance of using complete genomes for taxonomy of *Aeromonas.* The *A. hydrophila* strains were classified into two distinct phylogenetic groups (Figure 1). Group 1 contained strains isolated from diseased fish, except pc104A, which was recovered from the soil of a catfish pond that experienced an epidemic outbreak of septicemia caused by *Aeromonas* (Pang et al., 2015). Most of the strains were related to epidemic outbreaks of motile *Aeromonas* septicemia in catfish that occurred in the United States (ML09-119, AL09-71, and pc104A) or carp in China (NJ-35, JBN2301, and J1), and share a recent common ancestor, as shown by comparative genomic analyses (Hossain et al., 2014). These strains were previously classified as hypervirulent *A. hydrophila* (vAh) based on phenotypic and genotypic tests or *in silico* analysis for pathotype-specific PCR of vAh isolates (Pang et al., 2015; Rasmussen-Ivey et al., 2016a).

Strains ZYAH72, D4 and GYK1, isolated from diseased fish in China, are also part of phylogenetic group 1. These United States and Chinese strains were separated into distinct subclades of group 1 (Figure 1), what is in agreement with the data of Pang et al. (2015).

Strains from phylogenetic group 2 were recovered from different sources and were also separated into two subclades based on their geographical origins (Figure 1), and the South Korean strain grouped together with the United States strains. Three of these strains (AH10, ATCC 7966, and AL06-06) were previously studied and considered to be non-virulent *A. hydrophila* (non-vAh) in relation to the hypervirulent vAh strains recovered from fish disease outbreaks (Pang et al., 2015; Rasmussen-Ivey et al., 2016a). Our data confirmed the results of other phylogenetic studies indicating the separation of vAh from non-epidemical strains (Pang et al., 2015; Rasmussen-Ivey et al., 2016a).

Strains from groups 1 and 2 differed in several characteristics, including genome size, number of predicted GIs, GI average size, and ratio of GIs relative to genome size (Table 2), in which strains from group 1 generally present greater numbers. Genomic GC content also varied among these strains, from 60.50% to 60.90% in group 1 and from 61% to 61.60% in group 2. Strains from groups 1 and 2 also differed in terms of the functions encoded on the predicted GIs, although some features were common among them (Table 3).

The distribution of predicted GIs containing the main functions identified, and some features whose function have yet to be demonstrated in *A. hydrophila*, varied among the strains (Table 3), while some were common and other were encoded in a few strains. Among the identified characteristics are clusters for the synthesis of O Ag. O Ag, the most surface-exposed part of the lipopolysaccharide, a component of the outer membrane of Gram-negative bacteria, mediates pathogenicity by protecting infecting bacteria from serum complement killing and phagocytosis. It consists of repeating oligosaccharide subunits whose variability confers immunological specificity (Tomás, 2012). Several O Ag gene cluster types have been described in *A. hydrophila* (Hossain et al., 2013; Pang et al., 2015; Cao et al., 2018) and previously associated with GIs (Hossain et al., 2013; Pang et al., 2015). Gene clusters for O Ag were found in the predicted GIs of all strains; however, they differed in their gene content and were related to 10 distinct serogroups (Figure 2).

Thirteen of the strains analyzed in this study had their O Ag gene clusters previously determined (Hossain et al., 2013; Pang et al., 2015; Cao et al., 2018).

The putative serogroup OX1 was proposed for strains ML09-119, AL09-71, pc104a, JBN2301, and D4 strains (Cao et al., 2018). Strain ZYAH72, which had no previously analyzed O Ag cluster, shared the same cluster with the strains above (Figure 2). Thus, it should also be included in the putative OX1 serogroup. Strains NJ-35 and GYK1 were classified as putative serogroups OX6 (Cao et al., 2018). According to our data, strain J-1 should also be included in this serogroup because it shares the same O Ag biosynthesis cluster. This is in agreement with Pang et al. (2015), who showed that J-1 and NJ-35 have identical O Ag gene clusters.

All other strains had unique O Ag clusters. In contrast to the vAh fish pathogens that presented putative OX1 or OX6 O Ag clusters, strains AL06-06 and AH10 were also recovered from diseased fish but were considered as non-vAh (Rasmussen-Ivey et al., 2016a) and presented clusters OX4 and OX5, respectively (Cao et al., 2018). Strain ATCC 7966 belongs to the O1 serogroup (Seshadri et al., 2006), AHNIH1 to O25, WCHAH045096 to OX7 (Cao et al., 2018). The ZYAHA75 O cluster is similar to O18, and those of MX16A and KN-MC-1R2 were not determined (Figure 2).

Thus, no O Ag gene cluster was shared among strains from phylogenetic groups 1 and 2. All strains from group 2 exhibited unique O Ag clusters. Of the 10 distinct serogroups detected, only OX1 and OX6 were shared, and the identification of these clusters might be an alert for potential virulent strains related to outbreaks of epidemic diseases in fish.

Several genes associated with polar flagella biogenesis and chemotaxis were identified in the predicted GIs of most strains (Table 3; Supplementary Table 3). Mesophilic *Aeromonas* have a polar flagellum for swimming in liquid, and some strains can also express multiple inducible lateral flagella for swarming over viscous environments or surfaces (Janda and Abbott, 2010). The polar flagellum genes are clustered in five distinct regions, whereas lateral flagella genes are organized in only one cluster (Tomás, 2012). Beyond their role in motility, in *Aeromonas* flagella act as adhesins for human intestinal enterocytes and in biofilm formation (Kirov et al., 2004), and are essential for adhesion and the ability to invade fish cell lines (Beaz-Hidalgo and Figueras, 2013). In the current study, GIs containing gene clusters for polar flagella region 2 (*flaA flaBGHJ maf-1*) (Canals et al., 2006) were predicted. However, the data indicated that gene content downstream of polar flagella region 2 is heterogeneous in different strains (Supplementary Table 3) and that genes associated with flagella glycosylation, such as *flmD* and *neuB* (Tabei et al., 2009; Wilhelms et al., 2012; Fulton et al., 2015) are adjacent to region 2 in certain *A. hydrophila* isolates. These data are in agreement with a recent study (Forn-Cuní et al., 2021) that showed that *pse/flm* genes (*flmA flmB neuA flmD neuB*) related to polar flagellin glycosylation are clustered in highly polymorphic GIs with three main genomic glycosylation islands identified.

Groups I and III contain almost exclusively the *pse* genes (*flmA flmB neuA flmD neuB*). *Aeromonas* belonging to these groups have flagellins modified with a single monosaccharide pseudaminic acid derivative (Forn-Cuní et al., 2021). Strains from phylogenetic group 1 and strain AH-10 belong to the glycosylation genomic island group I, while MX16A and WCHA045096 were included in group III according to Forn-Cuní et al. (2021).

The glycosylation GI group II contains other genes between the *pse/flm* genes, and are present in strains that modify polar flagellins with heterogeneous glycan moieties. Strains ATCC 7966, AL06-06, and AHNIHI are part of this group together with KN-Mc-1R2 and ZYAHA75 (Forn-Cuní et al., 2021). Flagellin glycosylation is essential for flagellar function (Tabei et al., 2009) and polar flagellar glycan composition can differ between *A. hydrophila* strains (Wilhelms et al., 2012; Fulton et al., 2015; Forn-Cuní et al., 2021). Because *Aeromonas* flagella have several roles in pathogenesis (Kirov et al., 2004; Beaz-Hidalgo and Figueras, 2013), based on data showing that strains ML09-119 and AL09-71 (pathogens of catfish), AH10, J-1, JBN2301, and NJ 35 (pathogen from carp) share the same predicted GIs related to flagellar region 2, and results from Forn-Cuní et al. (2021), showing that they belong to the glycosylation GI group I, we speculate that flagellin glycosylation patterns may favor interactions with determined hosts.

VgrG and Hcp play roles in *Aeromonas* virulence, influencing bacterial motility, protease production, and biofilm formation, inducing host cell toxicity, apoptosis, and activation of macrophages (Suarez et al., 2008, 2010a,b; Sha et al., 2013). They are components of the T6SS which in addition to virulence plays roles in inter-bacterial competition. T6SS is encoded within gene clusters containing the core genes of the secretion machinery, however, additional copies of *hcp* and *vgrG* can be found outside the main cluster (Cianfanelli et al., 2016; Moriel et al., 2021), which explains the presence of these genes in two distinct predicted GIs in certain strains (Supplementary Table 3). However, a search in the genome showed that strain AL06-06 does not have the complete T6SS cluster, differently from strains AH10 and KN-Mc-1R2. This is in agreement with Tekedar et al. (2019) which showed that this strain have only three components of T6SS, *hcp, tssH* and *vgrG*. In their study they also showed that virulent strains including ML09-119, AL09-71 and pc104A also have only these T6SS components, and that *hcp1* and *vgrG1* contribute for virulence of the ML09-119 in catfish.

Some other genes with functions identified in predicted GIs (Supplementary Table 3) are associated with virulence in other bacteria, but their roles in *Aeromonas* have yet to be determined. Among them are the zonula occludens toxin and the accessory cholera enterotoxin, which have been described in *Vibrio cholerae* and have the ability to affect intestinal function in rabbits (Trucksis et al., 1993; Fasano et al., 1997). Another is the ankyrin protein, AnkB. In plant-pathogens *Xanthomonas oryzae* pv. oryzae and *X. oryzae* pv. oryzicola, AnkB participates in biofilm formation, swimming ability, exopolysaccharide production, and defense against oxidative stress. In these bacteria, the *ank*B gene is located 58 bp downstream of *catB*, which encodes the enzyme catalase. AnkB affects *catB* gene expression, catalase activity, and sensitivity to H_2_O_2_ (Pan et al., 2018). In *A. hydrophila*, the *ankB* gene is in tandem with *katE*, which encodes catalase, and in most strains with a gene encoding a superoxide dismutase precursor. Although *A. hydrophila* is an opportunistic pathogen in animals, this led us to speculate regarding its roles in defense against oxidative stress, as shown in *X. oryzae*.

Fic proteins are widespread in bacteria and are often found encoded in GIs. They may play a role as toxins secreted from pathogens, or as the toxin component of toxin/antitoxin modules. However, the great majority of Fic proteins have unknown functions (Veyron et al., 2018).

Several genes from the core genome were found in the predicted GIs (Supplementary Table 3), which should be further considered. Except for the *scsABCD* locus associated with copper resistance, which was present in the predicted GIs of all strains analyzed, the distribution of GIs encoding resistance-associated features was limited to four strains in phylogenetic group 2 (Table 3). The full *scs* operon appears to be limited to some Enterobacteriaceae (*Salmonella enterica*, *Serratia proteamaculans*, *Citrobacter koseri*, and *Klebsiella pneumoniae*), *Aeromonas hydrophila*, and *Photobacterium profundum* (Subedi et al., 2019). The *scsABCD* locus was originally identified in *S. enterica* Typhimurium, and is required to deal with copper and H_2_O_2_ stress, helping to increase *Salmonella* survival under severe copper and oxidative stress, hostile conditions encountered by the pathogen during its intracellular survival (López et al., 2018) thus, contributing to virulence. However, copper may also be found in water (Domek et al., 1984). Considering that *Aeromonas* are ubiquitous in aquatic habitats and are considered as opportunistic pathogens (Janda and Abbott, 2010), potential roles of *scsABCD* may contribute to fitness in environment and virulence in the host.

In *A. hydrophila*, *scsSBCD* is located in the predicted GIs distinct from those encoding features associated with resistance (Supplementary Table 3).

*Aeromonas* have intrinsic resistance to some β-lactams due to the production of one to three inducible β-lactamases. The resistance phenotypes vary in relation to the species according the production of one to three inducible β-lactamases, with five basic phenotypes described (Fosse, 2010). In *A. hydrophila* three classes of β-lactamases, a class B metallo-β-lactamase, a class C cephalosporinase, and a class D oxacillinase were described (Fosse, 2010; Janda and Abbott, 2010), conferring to the species the penicillinase-cephalosporinase-carbapenemase phenotype exhibiting resistance to ampicillin, amoxicillin, ticarcillin, cephalothin, and profile susceptible or intermediate to imipenem (Fosse, 2010). We looked for genes encoding these beta lactamases and subclass B2 metallo-β-lactamase (CphA family, or ImiH or ImiS), cephalosporin-hydrolyzing class C β-lactamase (CepS or CepH) and OXA-12 family class D β-lactamase (AmpH/OXA-724 or AmpS/OXA-725 or OXA-726) were identified in the chromosome of all 17 strains. Additionally, a tetracycline efflux MFS transporter TetE was identified in the chromosome of 2 strains.

*Aeromonas* are usually susceptible to aminoglycosides, tetracyclines, chloramphenicol, sulfonamides, trimethoprim, nitrofurans, nalidixic acid, fluoroquinolones (Fosse, 2010), carbapenems, third- and fourth-generation cephalosporins, macrolides, monobactams and extended spectrum penicillins (Janda and Abbott, 2010; Fernández-Bravo and Figueras, 2020). However, strains presenting resistance to one or more of these antibiotics are common in clinical and environmental strains (Aravena-Román et al., 2012; Esteve et al., 2015). Studies on molecular antibiotic resistance have shown that class 1 integrons, involved in intraspecific and interspecific dissemination of resistance, and large plasmids are associated to resistance to several antibiotics in *Aeromonas* (Nguyen et al., 2014; Hughes et al., 2016).

*Aeromonas hydrophila* multidrug-resistant strains are frequent, mainly among clinical isolates (Esteve et al., 2015; Hughes et al., 2016). Among the strains analyzed, the following contains plasmids, as informed at the NCBI database: JBN2301 (3 plasmids), D4 (4 plasmids), AHNIH1 (1 plasmid), AL06-06 (3 plasmids) and WCHAH045096 (6 plasmids). Plasmids from strains JBN2301and AL06-06 do not contain resistance genes. In strain D4, only one of the plasmids contain a gene encoding a quinolone resistance pentapeptide repeat protein QnrS2. The plasmid of strain AHNIH1, contains several genes encoding for resistance, as described (Hughes et al., 2016). Includes three class A beta-lactamases (KPC-2, SHV-12 and CARB-12) and genes encoding for resistance to aminoglycosides, chloramphenicol, fluoroquinolones, macrolides, sulfonamide and trimethoprim. Three of the plasmids of WCHAH045096 carry resistance genes. They are associated to the resistance to several classes of antibiotics including aminoglycosides, sulfonamide, trimethoprim, quinolone, tetracycline and carbapenem.

Predicted GIs containing antibiotic resistance associated genes had heterogeneous distribution among the *A. hydrophila* (Supplementary Table 4), possibly reflecting horizontal gene transfer and selection with the exposure to antibiotics in their environments. These predicted GIs were identified in four strains, recovered from water, sewage, fish or human, indicating that antibiotic resistance associated with GIs is spread in clinical and environmental strains. Most of these predicted GIs also encode transposases, integrases, and mobile elements, and one of them also includes proteins related to conjugation (Supplementary Table 3). In the strain AL06-06, a predicted GI encodes for aminoglycoside, sulfonamide, chloramphenicol and tetracycline resistance. The predicted GI of WCHAH045096 encodes for resistance to the same antibiotics described above, and contains genes for several proteins associated with mercury resistance. The predicted GI of strain MX16A encodes antibiotic resistance for tetracycline, aminoglycoside, chloramphenicol, sulfonamide, macrolide and beta-lactams e proteins related to mercury resistance. Strain ZYAH75 has three predicted GIs containing resistance genes. One of them contain genes for resistance to aminoglycoside and sulfonamide together with proteins for mercury resistance. This predicted GI is a mosaic of proteins associated with mobile elements, it encodes conjugative transfer proteins TrbJ, TrbK and TrbK, IncP-type oriT binding proteins TraJ and TraK, together with integrase, transposase, IS and mobile element proteins. Genes for chloramphenicol and aminoglycoside resistance are encoded in two other islands, which contains additionally genes encoding class A extended-spectrum beta-lactamase CTM-3, class A broad-spectrum beta-lactamases TEM-1, and gene for sulfonamide resistance; or genes for resistance to macrolides and quinolone, oxacillin-hydrolyzing class D beta-lactamase OXA-10, and also proteins associated with mercury resistance. Integron integrase Intl1 are encoded in two of the predicted GI of ZYAH75, and those from strains WCHAH045096 and MX16A. In several of these predicted GIs there is more than one gene encoding for aminoglycoside modifying enzymes, and for sulfonamide resistance. Therefore, in addition to the intrinsic resistance characteristic of the species, some strains analyzed contain determinants for resistance to tetracycline in the chromosome (2 strains); plasmids carrying genes for resistance to one and up to six distinct classes of antibiotics, and even more than one plasmid related to resistance, and predicted GIs in which several genes of resistance are encoded, the most common are those related to the aminoglycoside and sulfonamide resistance. *Aeromonas* are ubiquitous in aquatic environments and widely distributed, being found in every environmental niche where bacterial ecosystems exist, facilitating the contact with humans (Janda and Abbott, 2010). Thus, the presence of GIs carrying resistance genes in *Aeromonas* may impact the fitness of the bacteria, facilitate the resistance dissemination, and spread of resistant strains in several environments.

Some of the predicted GIs shared among strains from phylogenetic group 1 encode metabolic pathways for utilization of myo-inositol and L-fucose/sialic acid, which were previously described in GIs of *A. hydrophila* strains NJ-35, J-1, ML09-119, AL09-71, and pc104A (Pang et al., 2015). In the current study, these features were also found in the predicted GIs of other strains of group 1. These pathways may be linked to the full virulence of fish disease epidemics *A. hydrophila* (Pang et al., 2015), suggesting that these GIs may be related to fitness or enhancing adaptation to hosts and competitiveness.

Other predicted GIs common to vAh contain genes encoding QueD, QueE, and QueC (Supplementary Table 3), which participate in the synthesis of PreQ_0_, a precursor of queuosine- (Q) tRNA modification in bacteria. PreQ_0_ is reduced to PreQ_1_ and inserted in the G residue at position 34 of tRNAs with GUN anticodons by tRNA guanosine transglycosylase (bTGT); however, the enzyme can also use PreQ_0_ when PreQ_1_ is absent (Thiaville et al., 2016). However, predicted *A. hydrophila* GIs encoding QueDEC also encode TgtA5, a variant of TGT that has divergent features from bTGT, helicases, and proteins involved in DNA repair (Supplementary Table 3). Similar clusters have been described in a GI of *Salmonella enterica* serovar Montevideo and by comparative genomic analysis in several other bacteria, including *A. hydrophila* ML09-119. In *S. enterica* serovar Montevideo, *Kineococcus radiotolerans*, *Comamonas testosteroni*, and *Sphingopyxis alaskensis*, clustering of tgtA5 and preQ_0_ synthesis genes is involved in inserting 7-deazapurine derivatives into DNA, suggesting a new and complex system of DNA modification (Thiaville et al., 2016). The SAM-dependent methyltransferase HI0095 (UbiE paralog), which is associated with virulence in vAh (Rasmussen-Ivey et al., 2016a) is also encoded in this predicted GI. Thus, the roles of these genes in *A. hydrophila* DNA modification and/or virulence have to be determined.

No distinctive characteristics of strains from group 2 were identified, and most isolates shared only a small number of predicted GIs (Supplementary Table 6), suggesting a high degree of strain diversity. Of note, several of these strains have multiple determinants of antimicrobial resistance encoded by their predicted GIs. Group 2 contains only eight strains; however, they may better reflect the diversity of GIs in *A. hydrophila* because previous studies have shown that most strains from group 1 are highly related and constitute a clonal group (Hossain et al., 2014; Pang et al., 2015; Rasmussen-Ivey et al., 2016a).

### Addendum

At the end of this study, new complete genomes of *A. hydrophila* were available in the NCBI database reaching a total of 40, of which 19 were previously analyzed. Information on the new 21 strains is showed in Supplementary Table NST1. A complementary phylogenetic analysis including all 40 genomes indicated that four of the strains classified as *A. hydrophila* were misidentified at species level; two are the new strains NEB724 and B11, and YL17 and 4AK4 which were previously excluded from our study (Supplementary Figure 1). The 36 *A. hydrophila* were separated in two main branches (Supplementary Figure 1). However, one branch contains only new strains isolated from chicken (4 strains). The other branch includes subgroups containing the strains previously classified in group 1 (Vah) plus new isolates from snakes (2), fish (1) and without info (1); and the strains from previous group 2, in which new isolates from water (4), fish (4), human (1), and no source info (2) were included (Supplementary Figure 1). The raw data of Island Viewer4 analysis performed with the new genomes are showed in Supplementary Table NST2. Among the 19 new genomes of *A. hydrophila*, 735 GIs were predicted, however, these GIs were not manually curated. Gene clusters for O Ag biosynthesis were identified in predicted GIs. The relationship among the O antigen gene clusters of the 36 *A. hydrophila* was analyzed through nucleotide sequences alignment performed using the Clustal Omega Web Server^3^, with “default” parameters and the software IQ-TREE - Efficient Tree Reconstruction (version 1.6.12 – Windows 64; Nguyen et al., 2015, Mol. Biol. Evol., 32:268–274, 2015), which was used to build the phylogenetic tree of the clusters. The parameters used were “default” for ModelFinder, with bootstrap and maximum likelihood values. Results are indicated in the (Supplementary Figure 2), which indicates that two of the new strains present O Ag cluster already identified among the strains previously analyzed. Strain LHW39 presents the OX1cluster which was observed among some of the VAh, and strain HX-3 the OX-5 cluster, identified in strain AH10. Fourteen O Ag cluster, apparently distinct of those identified among strains previously analyzed, were found in GIs of 17 new genomes (Supplementary Figure 2); similar O Ag clusters were found in GIs of strains 23-C-23 and WCX23-1; Brac6 and ONP3-1; GSH8-2 and WP8-S18.ESBL02. Predicted GIs containing resistance genes were found in 5 strains (Supplementary Table NST3), and are related with resistance to tetracycline (4 strains), beta-lactam (class A extended-spectrum beta-lactamase CTX-M-14; 1 strain), macrolide (2 strains), aminoglycosides and sulfonamide (1 strain). The number of resistance features found in the predicted GIs of the new genomes varies from 1 to 3, in contrast with those observed in the previous analyzes which range from 3 to 9. These preliminary results confirm the previous analyzes indicating that some features, such as O Ag clusters, are associated to GIs in all strains. Features encoded on predicted GIs of the new genomes confirms the contribution of these elements for the diversity and antimicrobial resistance of *A. hydrophila.*

## Data Availability Statement

The datasets presented in this study can be found in online repositories. The names of the repository/repositories and accession number(s) can be found in the article/Supplementary Material.

## Author Contributions

AS designed the study, collected and data analysis, and manuscript writing. JM did the funding acquisition, data evaluation, and original draft writing. RR did the conception and design of the manuscript. CD did the phylogeny analysis. DJ developed scripts and contributed to data analysis. CF-P did the data analysis and manuscript writing. GP reviewed and edited the manuscript. All authors contributed to the article and approved the submitted version.

## Conflict of Interest

The authors declare that the research was conducted in the absence of any commercial or financial relationships that could be construed as a potential conflict of interest.

## Publisher’s Note

All claims expressed in this article are solely those of the authors and do not necessarily represent those of their affiliated organizations, or those of the publisher, the editors and the reviewers. Any product that may be evaluated in this article, or claim that may be made by its manufacturer, is not guaranteed or endorsed by the publisher.

## Footnotes

1
https://www.ncbi.nlm.nih.gov/pathogens/antimicrobial-resistance

2
https://github.com/Microsoft/PowerBI-visuals-chord

3
https://www.ebi.ac.uk/Tools/msa/clustalo/
