# Supplementary material for: Prediction and Analysis in silico of Genomic Islands in Aeromonas hydrophila
Source: Front Microbiol. 2021 Nov 29;12:769380. doi: 10.3389/fmicb.2021.769380 (PMC8667584; doi:10.3389/fmicb.2021.769380)
Supplement: Supplementary file 2 [file Table_2.DOCX]

Supplementary Material

## Supplementary Figure 1





**Supplementary Figure 1.** Phylogenetic analysis including 21 new genomes and the previous 19 genomes of strains classified as *A. hydrophila*.

## Supplementary Figure 2





**Supplementary Figure 2.** O antigen gene clusters in the new predicted GIs of the 36 *A. hydrophila*, with bootstrap values.
